# Patterning-mediated supramolecular assembly of lipids into nanopalms

**DOI:** 10.1016/j.isci.2022.105344

**Published:** 2022-10-13

**Authors:** Samar A. Alsudir, Alhanouf Alharbi, Abdulaziz M. Almalik, Ali H. Alhasan

**Affiliations:** 1 National Center for Biotechnology, Life science and Environmental Research Institute, King Abdulaziz City for Science and Technology (KACST), P.O. Box 6086, Riyadh 11461, Saudi Arabia; 2 KACST-BWH/Harvard Centre of Excellence for Biomedicine, Joint Centers of Excellence Program, King Abdulaziz City for Science and Technology (KACST), P.O. Box 6086, Riyadh 11461, Saudi Arabia; 3 College of Science and General Studies, Alfaisal University, P.O. Box 50927, Riyadh 11533, Saudi Arabia

**Keywords:** Chemistry, Supramolecular chemistry, Materials science, Biomaterials

## Abstract

At nanoconfined interfaces, a micellar ink of lipids was programmed to transform into various secondary structures such as discs, sheets, or sheet and discs via patterning-mediated self-assembly facilitated by polymer pen lithography. Nanoconfinement with printing force, humidity, temperature, pattern size, and total printing time all governed the intramolecular assembly of lipids and determined their structural shape and size. For example, disc or sheet architectures self-organized to produce cochleates or discotic liquid crystals, respectively. In contrast, the combined structure of sheet and discs produced a novel supramolecular output referred to as “nanopalms”. The mechanism of nanopalms formation and the origin of their stability were investigated and discussed. Post patterning treatment helped to transform the patterned discs into ribbons and sheets into cochleates that could facilitate the curling of ribbons along their folding direction in a programmed manner via intermolecular self-organization producing the nanopalms.

## Highlights

• Patterning of lipids offers a new route for generating novel supramolecular structures
• Nanoconfinement enables shape control over lipid assemblies
• Mechanism of nanopalm formation was proposed with the rules governing its stability

## Graphical abstract

**Figure:**
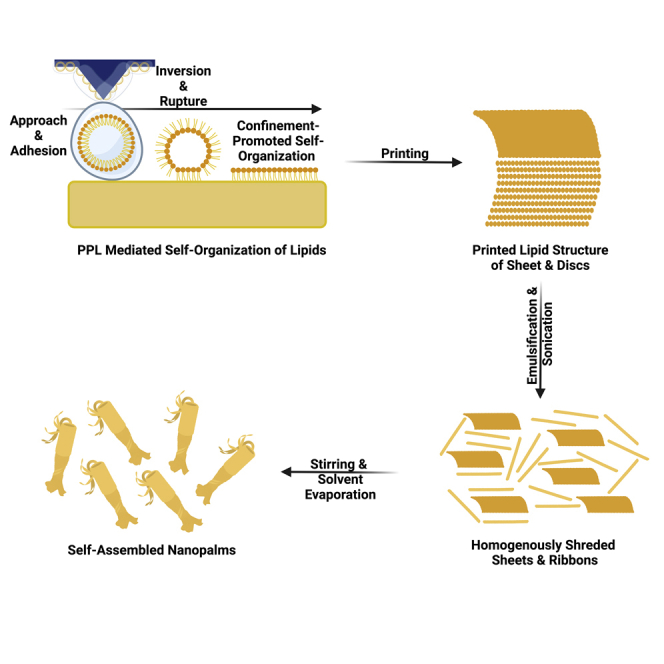

## Introduction

Self-assembly is a fundamental bottom-up process inspired by nature and used for the formation of higher-ordered structures starting from liquid disordered phases (Montis et al., 2021). Although intramolecular self-assembly produces well-defined secondary and tertiary structures out of polymers, instabilities exist within the corresponding lipid structures (Hecht, 2005) owing to their molecular dynamics, which is often dictated by lipid compositions and/or lipid geometry when present in liquid environments (Frolov et al., 2011; Tan et al., 2019). The ability of lipids to undergo supramolecular assemblies to form quaternary structures out of secondary and tertiary structures via intermolecular self-assembly might govern the formation of highly stable lipid structures, which are increasingly adapted for a variety of medical products ranging from organs’ self-targeting for accelerating clinical trials (Alsudir et al., 2021), to mRNA delivery (Pfizer, 2012; Moderna, 2021), and CRISPER-Cas9 *in-vivo* gene editing (Gillmore et al., 2021). Under suitable conditions, lipids could self-assemble into various nano-/micro-/mesoscale structures including micelles, spherical vesicles, nanotubes, nanoribbons (planar, undulating, and helical), cochleates, or discotic liquid crystals (Shanmugam and Banerjee, 2011). Papahadjopoulos *et al* were the first to discover cochleates in 1975 on observing bulky fusion and structural rearrangement of the unilamellar negative phosphatidylserine vesicles after Ca^2+^ addition resulting in their electrostatic attractions and subsequent transformation into large sheets. To minimize their interactions with the surrounding water molecules, sheets could fold spirally to form tertiary structures known as lipid cochleates (Papahadjopoulos et al., 1975). Besides sheets, ribbons are another secondary structure that could encounter curling, adhesion, or fusion to form cochleates due to their large energetic unfavorable surfaces (Nagarsekar et al., 2016). Variously, discotic liquid crystals constitute of multichain lipids such as triglycerides that can self-organize into Y-conformation with fully splayed chains (120° apart) on increasing temperature or addition of non-polar solvents. These splayed chains are loosely bound within disc-like structures that self-assemble via “interdigitated” stacking forming flexible columns/cylindrical rods (Corkery et al., 2007). Apparently, the molecular self-assembly of lipids to form secondary and tertiary structures is fairly understood; yet, a considerable portion concerning the spatiotemporal formation of hierarchical supramolecular lipid structures out of secondary and tertiary structures via intermolecular self-assembly remains unexplained.

Patterning of lipids on solid substrates in a spatiotemporally ordered fashion could offer an ideal opportunity for studying quaternary lipid structures. Polymer pen lithography (PPL) has been successfully used for fabricating patterned features made of hard matter such as metals (Chaia et al., 2010) or soft matter including proteins (Huo et al., 2008), carbohydrates (Bian et al., 2012), oligonucleotides (Kumar et al., 2016; Rani et al., 2019), and lipids (Kumar et al., 2017) rendering it potentially useful in advancing the field of supramolecular chemistry. A typical polymer pen array contains thousands of pyramidal elastomeric pens made of polydimethylsiloxane (PDMS) (Odom et al., 2002) to deliver different inks to the desired substrate facilitating the development of exquisite patterning capabilities such as multiplexing (Zheng et al., 2009; Brinkmann et al., 2013) over cm^2^ areas with sub-100 nm resolution in a high- throughput manner. Features on the nanometer and micrometer length scales can be fabricated owing to the time and force dependent-ink transport (Liao et al., 2010), expanding PPL tunability within the same pen array when brought into contact with the substrate after automated electrical leveling to ensure uniform pattern development. Combinatorial libraries having a tunable gradient of feature sizes can also be fabricated using individual pens by varying either the dwell time or the relative z-piezo extension, or by deliberately tilting the pen array relative to the substrate (Giam et al., 2012a, 2012b; Eichelsdoerfer et al., 2013). PPL has proven to be a powerful strategy for synthesizing complex, polyelemental nanoparticles by patterning polymeric features loaded with metal precursors, so-called nanoreactors, and transforming them into hard structures of metallic nanoparticles *via* post-patterning gaseous heat treatment (Giam et al., 2012a, 2012b; Chai et al., 2012; Kluender et al., 2019). However, generation of soft nanomaterials through PPL-defined nanoconfinement remains unexplored.

**Scheme 1:**
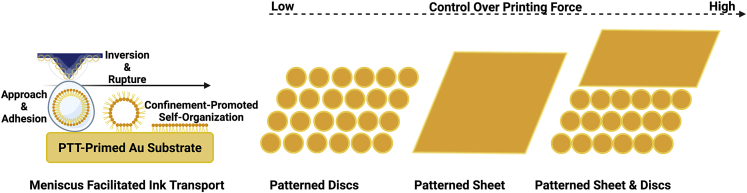
Force-mediated printing of patterned discs, sheet, sheet& discs.

Inspired by PPL capability to produce alloy nanoparticles out of miscible or immiscible metals owing to their physical nanoconfinement in patterned nanoreactors (Chen et al., 2015, 2017), we explored the effect of nanoconfinement of patterned lipids on promoting their self-assemblies into various secondary structures when they are forced into close proximity. Accordingly, we developed a PPL approach to self-organize a novel quaternary lipid structure; we referred to as “nanopalms”, each of which is anticipated to be a supramolecular product of the self-assembled secondary structures; sheets and ribbons. We hypothesize that controlling printing force can result in fabricating patterns of various structures including discs, sheets, and sheet and discs when delivering a micellar ink of lipids under suitable conditions to the 1-pentanethiol (PTT)-primed gold (Au) substrate as shown in Scheme 1. Subsequent sonochemical treatment of the developed “sheet and discs” structures with mechanical agitation and solvent evaporation enabled their self-organization into lipid nanopalms.

## Results

### Substrate hydrophobization and pen array inking design

**Figure 1:**
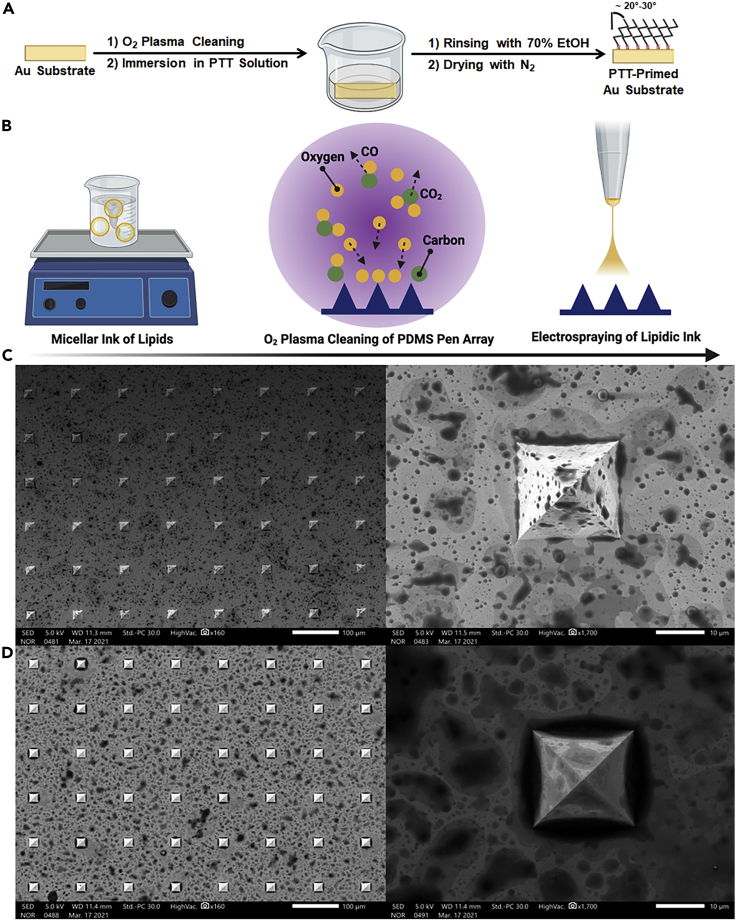
Preparation of PTT-primed Au substrate and the inked-pen array (A–D) Schemes of (A) Au substrate priming with PTT and (B) PDMS pen array inking with the micellar ink of lipids (created by BioRender). SEM Characterization of (C) inked pen array via electrospraying with scale bar 100 μm (left) and 10 μm (right), (D) inked pen array after humidification for 2 hours with scale bar 100 μm (left) and 10 μm (right).

Au substrates were plasma cleaned to remove organic contaminants and primed with PTT ethanolic solution under static vacuum at ambient conditions for 24 hours (Figure 1A). PTT molecules could self-assemble on Au substrates forming monolayers arranged in trans-conformation with a tilt angle of nearly 20–30°, which is known for the self-assembled *n*-alkanethiols on Au surfaces (Ulman, 1996; Lingler et al., 1997; Love et al., 2005). PTT modification rendered Au substrates with hydrophobic properties affecting the spreading behavior of the transferred ink onto the substrates. A micellar ink of lipids was formed in an aqueous solution because the chosen lipid concentrations were above their critical micelle concentration (CMC) (Cui et al., 2014), which is summarized in Table 1, with other factors that could affect the final packing organization into micelles including, but not limited to, temperature, the presence of surfactants, and the used solvent. The areas of hydrophilic head groups in lipids increase with temperature owing to the increased steric repulsion among them, and this decreases the value of the packing parameter, which is a metric that links lipid geometrical properties with their preferred packing organization (Israelachvili, 2011). The temperature used during ink preparation was 50°C, which could lower the value of the packing parameter to a certain extent. Also, the incorporation of the non-ionic Pluronic F68 surfactant, which is known to self-aggregate in aqueous solutions forming spherical micelles, can reduce the overall packing parameter. Studies confirmed the transformation of lipid vesicles into micelles by adding nonionic surfactants (Elsayed and Cevc, 2011; Mkam-Tsengam et al., 2022). Decreasing the value of packing parameter (≤0.33) could result in the formation of mixed micelles. Moreover, ethanol (EtOH), used originally to solvate the lipids, could intercalate and form hydrogen bonds with phospholipids’ head groups, decreasing the order in the lipid hydrocarbon chains. This increased disorganization enhances the lipid bilayer fluidity and thereby affecting the final packing organization into micelles (Patra et al., 2006; Toppozini et al., 2012).

**Table 1:** Critical micelle concentrations of phospholipids used throughout the study

Phospholipid	CMC (μM)	
	Room Temperature	45°C
DOPC	50	20
DOPE	800	200

Simultaneously, PDMS pen arrays were plasma cleaned to promote ink wetting before electrospraying of the micellar ink solution (Figure 1B). This inking strategy was preferred over spin coating and drop casting (Kluender et al., 2019; Chen et al., 2015, 2017; Ulman, 1996; Lingler et al., 1997; Love et al., 2005; Liu et al., 2020) because it resulted in the formation of singular and isolated micelles under specific process parameters facilitating the uniform ink coverage and thereby promoting the printing process via PPL (Figure 1C). In a typical experiment, the inked pen array was first mounted in the instrument and next incubated at a moderate temperature around 32 ± 2°C and a relative humidity (RH) of 92 ± 2% for 2 h before the actual printing process. This step is necessary to enable ink hydration, spreading, and diffusion thereby facilitating its transport to the substrate. Figure 1D shows the synergetic effect of temperature and humidity on ink spreading with pens coating. Phase transition temperatures (T_m_) of 1,2-dioleoyl-sn-glycero-3-phosphocholine (DOPC) and 1,2-dioleoyl-sn-glycero-3-phosphoethanolamine (DOPE) are −17°C and −16°C, respectively. Hence, their mixture with cholesterol would certainly be in the L_α_ liquid state at 32 ± 2°C. It is important to note that both DOPE and DOPC were carefully selected; DOPE was used to achieve the reverse micelles in an oily environment (the PTT-modified Au substrate) whereas DOPC is widely employed as a phospholipid ink in either dip pen lithography or PPL owing to its controllable diffusion by RH in the range of 40–95% under ambient conditions. At a RH higher than 70%, the phospholipid ink becomes sufficiently fluid and readily coats the tips (Lenhert et al., 2007; Urtizberea and Hirtz, 2015). Hence, temperature and humidity are considered to be the most critical parameters controlling ink mobility in a uniform fashion.

### Nanolithography printing design

Printing was carried out using PDMS pen arrays with 100 μm tip-spacing among the 40,000 pens at varied printing forces (150, 230, or 420 mN) and pattern sizes (30x30 or 40x40 dot matrix per one pen) while fixing the other instrumental parameters affecting the printing process including the applied voltage (5 V), current threshold (0.20 mA), dwell time (3 s), step size (0.7 μm), and the approach (100 μm/s) and retract velocities (30 μm/s). A temperature of 32 ± 2°C and a RH of 92 ± 2% were applied during the whole printing process.

**Figure 2:**
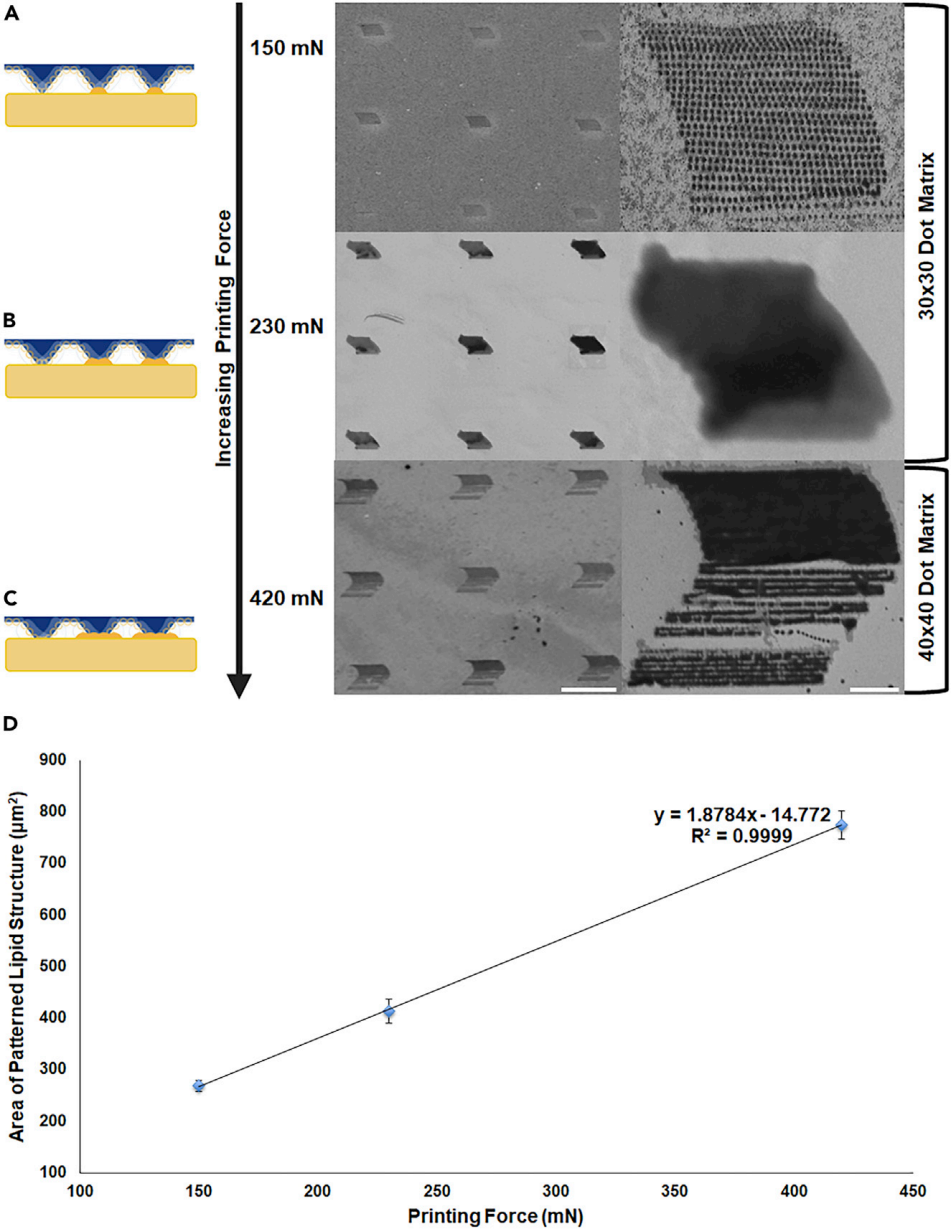
Varying the forces to form different lipid structures (A–D) Writing schemes at different forces and their corresponding patterned lipid structures (A) discs, (B) sheets, and (C) sheet& discs (Scale bars: 50 μm (left images); 10 μm (right images)). (D) Plot representing the area of patterned lipid structures (μm^2^) as a function of the printing force (mN).

On adhesion to the PTT-primed Au substrate, the printed lipid micelles tend to invert owing to the hydrophobic nature of the modified Au substrate (Scheme 1). The subsequent hydrophobic interactions between the inverted micelles and the substrate could possibly result in micelle deformation and rupture followed by self-assembly of lipid amphiphiles, which is a known mechanism for the dynamics of lipid vesicles on hydrophobic surfaces (Lingler et al., 1997; Ngassam et al., 2021). Resembling the molecular ink diffusive behavior (Brown et al., 2013), the phospholipid ink could diffuse in an isotropic fashion, adhere to the substrate, and subsequently self-assemble when forced into close proximity resulting in round printed features (discs) at 150 mN of printing force and a pattern size of 30×30 (Figure 2A). These results are in agreement with the previously reported data showing the round features of the printed phospholipids (Kumar et al., 2017; Urtizberea and Hirtz, 2015). These features tend to stack three dimensionally on the substrate owing to the slow spreading kinetics of the phospholipids; yet, they could spread laterally on hydrophobic surfaces to form a thin homogeneous layer when printing is carried out at high humidity (≥90%) and moderate temperature (26 ± 2°C) for extended periods of time (≥1 hr) (Lenhert et al., 2007; Urtizberea and Hirtz, 2015). Increasing the printing force to 230 mN (Figures 2B) and 420 mN (Figure 2C) resulted in increasing the tip-substrate contact area as well as the amount of delivered ink allowing the lipid discs to fuse forming other structures; sheets (pattern size of 30×30) and sheet and discs (pattern size of 40x40), respectively. Apparently, implementation of varied printing parameters within the same overall assembly program results in different self-organization processes and hence various lipid-assembled structures such as discs, sheets, or sheet& discs. Further SEM characterization of the patterned lipid structures can be viewed in Figure S1. Being in a very close proximity owing to the nanoconfinement effect with the applied force and humidity, printed discs tend to aggregate and their outer bilayers leaflets can subsequently merge with their internal contents permitting their intramolecular self-assembly. Within the force threshold, we can observe a linear relation between the printing force and the area of lipid structure at a fixed dwell time of 3 s and a RH of 92 ± 2% (Figure 2D). Apparently, generating structures of various areas and shapes is greatly affected by the force-dependent ink transport in parallel to the force and humidity-mediated feature self-assembly. When using the pattern size of 30×30, the area of patterned sheets printed at 230 mN became larger than that of patterned discs printed at 150 mN. Upon increasing the printing force to 420 mN with utilizing the pattern size of 40×40, the area of patterned sheet& discs became even larger.

Another way to obtain differently sized and shaped structures is by modulating the total time needed for printing, which relates to various parameters including dwell time, pattern size, and the best touch position (which is the z coordinate of the precise/piezo xyz motor on obtaining an electrical contact between the array and the substrate after automated electrical leveling). Fixing the dwell time while increasing the pattern size and having a longer time per point (relating to the approach and retract velocities and the set z-piezo touch position) could greatly increase the printing total time affecting the resulting shape and size. Indeed, we can generate differently sized and shaped structures by simply controlling the printing force and time. Table 2 summarizes the various experimental and instrumental parameters used for lipid ink printing.

**Table 2:** Various experimental and instrumental parameters used for lipid ink printing

Shape	Force (mN)	Pattern Size (Dot)	z-piezo touch position (μm)	Dwell time (s)	Step size (μm)	RH (%)	Total time of printing (hrs)
Discs	150	30×30	68	3	0.7	92 ± 2	2.00
Sheet	230	30×30	50	3	0.7	92 ± 2	1.75
Sheet& Discs	420	40×40	77	3	0.7	92 ± 2	4.00
Ribbons	450	40×40	64	3	0.7	92 ± 2	3.25

Another interesting lipid structure consisting of discs fused into ribbons (Figures S2A and S2B) was obtained with slight modification of the instrumental parameters used to obtain sheet and discs, which include the printing force (450 mN) and the z-piezo touch position (64 μm). Having a slightly shorter z-piezo touch position profoundly affected the total time of printing (3.25 hrs), which permitted the lateral fusion of discs into ribbons in a high humid environment (92 ± 2%). Indeed, total time of printing with high humidity could intensely alter the final shape of the obtained lipid structures. Hence, PPL provides exquisite control over the spatial and temporal features of lipids.

### Emulsion of the assembled lipid structures

**Figure 3:**
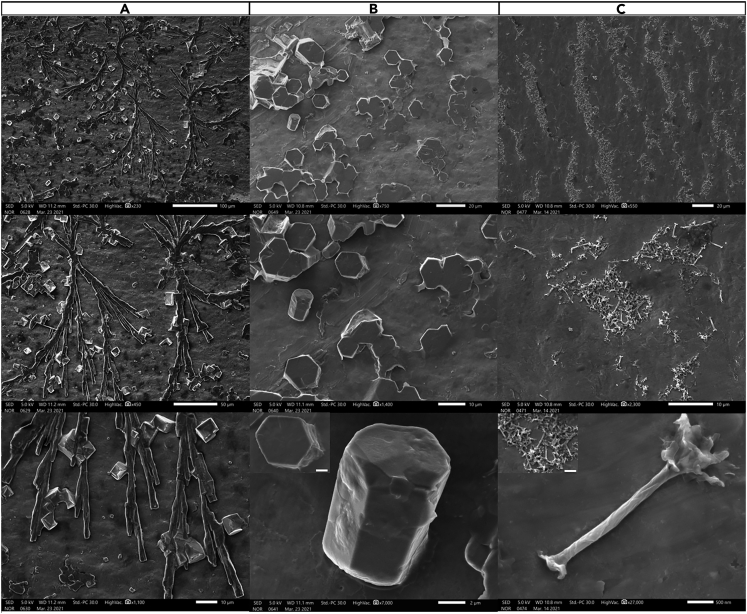
SEM micrographs of the generated supramolecular lipid structures (A–C) (A) cochleates, (B) discotic liquid crystals, (C) nanopalms at different scales; 100 μm-500 nm. Scale bar of insets at the bottom of panels (B and C): 2 μm.

**Figure 4:**
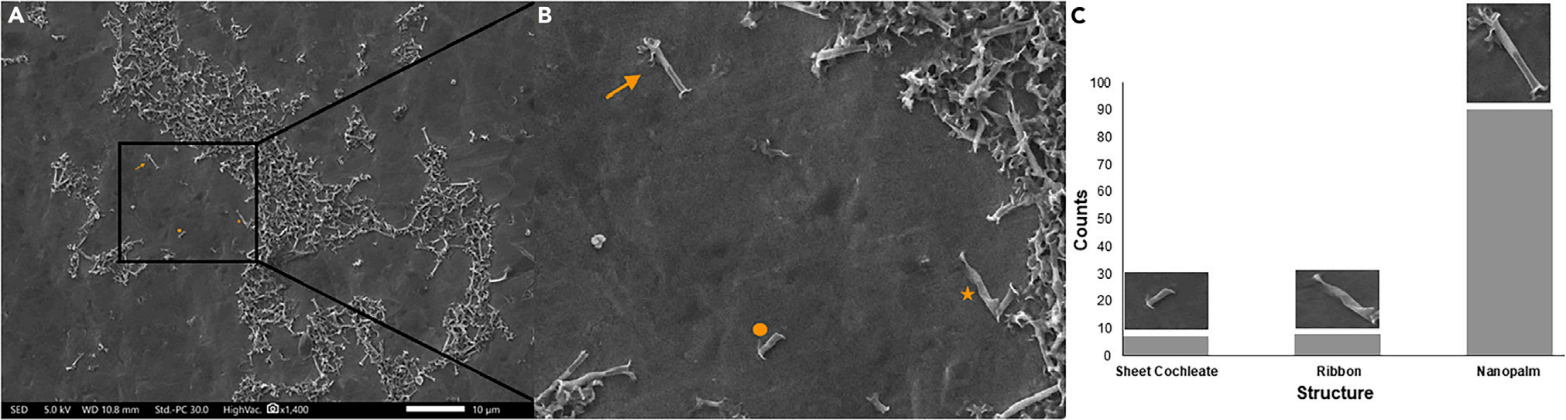
Structural characterization of nanopalms (A–C) (A) An SEM micrograph of nanopalms along with its constituents (SCs and ribbons), (B) a zoomed in view showing a SC (annotated with an orange circle), a ribbon (annotated with an orange star), and a nanopalm (annotated with an orange arrow), (C) a histogram of counts *vs* structure verifying the high structural uniformity of nanopalms.

Chemical treatment of the patterned lipid structures in a solvent mixture of chloroform CHCl_3_/EtOH/DDW (3:1:1) v/v% with sonication (10 min) and mechanical agitation (20 min) facilitated their transfer into the liquid phase and possibly shredding into smaller structures while preserving their original shapes. Fast evaporation of CHCl_3_ and EtOH at 50 ± 5°C under continuous stirring at 900 rpm for 48 hrs produced different supramolecular outputs with regards to the emulsified lipid structures. Well-known lipid structures such as cochleates (Figure 3A) or discotic liquid crystals (Figure 3B) could be generated owing to the hierarchical self-organization of discs or sheets, respectively, via intermolecular self-assembly. In a similar manner, emulsification of the combined structure of sheet& discs along with the slow evaporation of CHCl_3_ and EtOH (at 35±5°C under continuous stirring at 900 rpm for 48 hrs) produced a novel homogenous supramolecular structure, shape of which resembles the *Adonidia merrillii* (a.k.a. Christmas palms), and hence the nomenclature “lipid nanopalms” (Figure 3C). Each nanopalm consists presumably of multiple ribbons formed on discs fusion and wrapped around a sheet cochleate (SC), a tertiary structure, to form the tightly wrapped lower part while continuing to curl to form the loosely wrapped upper one. Ribbons and sheets are secondary structures produced via the intramolecular self-assembly among the printed discs, which was mediated primarily by force and humidity in confined spaces. Nanopalms, on the other hand, are supramolecular structures produced via the intermolecular self-assembly between SCs and ribbons in solution with the aid of magnetic stirring. A difference in contrast can be seen an all SEM images of nanopalms between the tightly and loosely parts representing the intermolecular self-assembly between SCs and ribbons. The average outer diameters of the tightly and loosely wrapped parts of the nanopalms were 206 ± 20 nm and 267 ± 25 nm, respectively whereas their entire length was around 3 ± 1 μm. Further SEM and TEM characterization of lipid nanopalms could be seen in Figure S3. Apparently, our developed PPL approach is greatly dependent on printing discs of high consistency with their consecutive secondary structures to generate different supramolecular entities of high uniformity. Careful analysis of Figure 4A, showed 7 SCs, 8 ribbons, and 90 nanopalms, which are clearly differentiated in Figure 4B. A histogram of counts versus structure (Figure 4C) was accordingly generated to present a strong evidence of the high structural uniformity of nanopalms that stemmed originally from the nanoconfinement of lipids via PPL, which resulted in printing sheet& discs structures of high consistency as shown in Figure 2C. Emulsification and solvent evaporation with mechanical agitation of these structures produced the nanopalms. Contrarily, emulsification of the ribbons structures resulted in random stacking as shown in Figure S2C owing primarily to the absence of chirality in the used phospholipids, which is the common driving force for ribbons curling.

### Role of nanoconfinement

**Figure 5:**
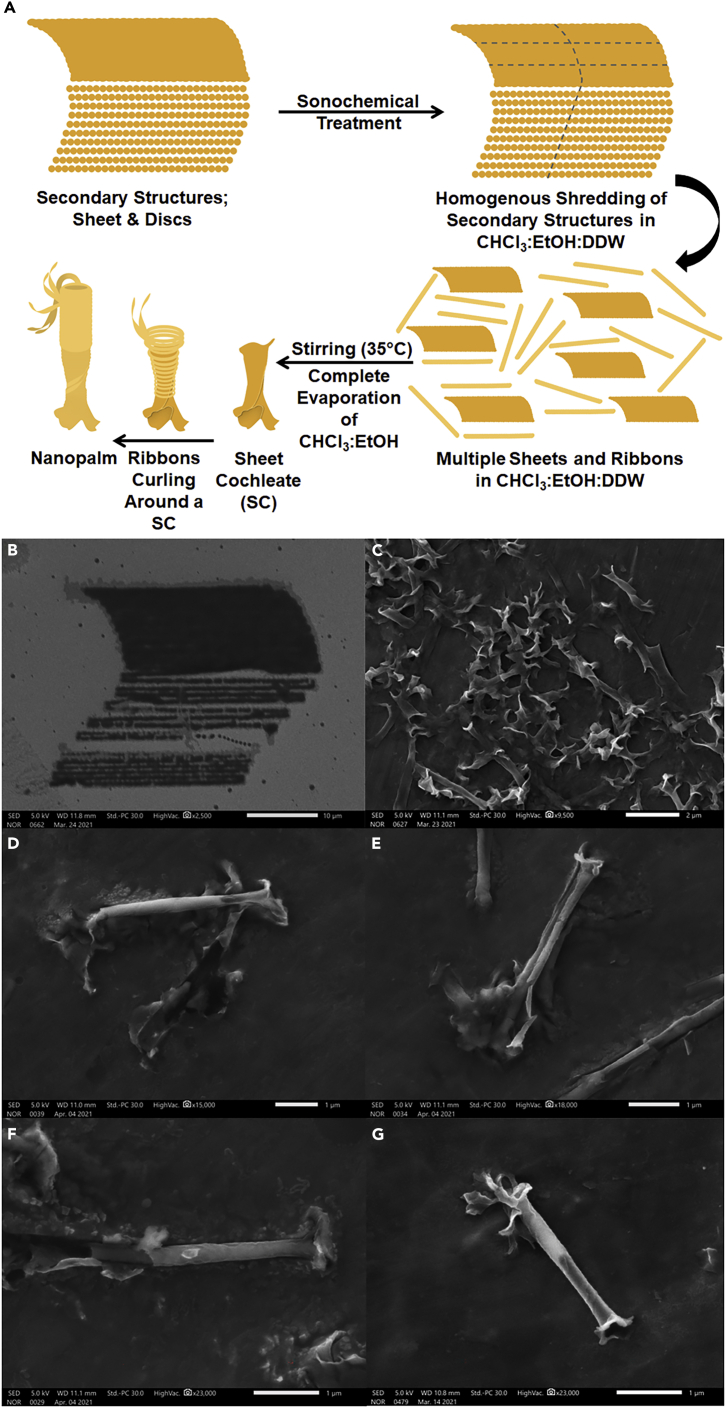
Structural deformation of nanopalms to investigate its mechanism (A–G) (A) Schemes for the proposed mechanism of lipid nanopalm formation. SEM characterization of (B) printed “sheet& discs” structure, (C) fully deformed nanopalms into sheets and ribbons after high-speed vortexing of a 3-weekold nanopalm suspension, (D, E and F) partially deformed nanopalms, (G) an intact nanopalm. The fully deformed and partially deformed nanopalms were all observed in one experiment from the same batch.

Molecular self-assembly has offered a prominent route for the generation of well-defined and discrete nano-/microstructures via programmed arrangement of amphiphilic molecules, which is mainly driven by electrostatic attractions and affected by molecular chirality, elasticity of orientational order, or spontaneous curvature (Selinger et al., 2001; Zhu et al., 2019). Hydrophobic interactions, H-bonding, and van der Waals attractive forces could also drive the self-assembly of amphiphilic molecules when dispersed in aqueous environments at concentrations above their CMC (Barclay et al., 2014; Huang et al., 2017). Our findings suggest the ability to pattern various structures of lipids including discs, sheets, sheet and discs, or ribbons when lipid molecules are forced into close proximity. This facilitated the generation of well-defined morphologies that might not exist in nature such as cochleates, discotic liquid crystals, and nanopalms. Novelty of nanopalms steered the research direction toward investigating their mechanism of formation, which is proposed in Figure 5A. Emulsification and sonication of the sheet& discs structures (Figure 5B), then complete solvent evaporation triggered discs fusion into ribbons and sheet folding into SC (Figure S4) followed by ribbons wrapping the SC while continuing to curl to ultimately form a nanopalm. Such intermolecular self-assembly between the two secondary structures, sheets and ribbons, was most likely mediated by hydrophobic interactions and H-bonding. To shed more light into the mechanism of nanopalm formation, we analyzed their 3-weekold suspension after vigorous vortexing that was carried out for about a minute to forcefully deform the nanopalms owing to the high shear stress. Fully deformed nanopalms (Figure 5C) produced back their secondary/tertiary structures; sheets (unfolded or folded) and ribbons (uncurled or curled). Some nanopalms were partially deformed (Figures 5D–5F) revealing torn ribbons along their rolling direction exposing the inner SCs whereas the others were fully intact (Figure 5G). These results with the supporting TEM images shown in Figure S5 validate the proposed mechanism of nanoplam formation. Apparently, the formed SCs dynamize the curling of ribbons along their folding direction because chirality, which is the usual stimulant for ribbons scrolling, is absent in the used phospholipids.

**Figure 6:**
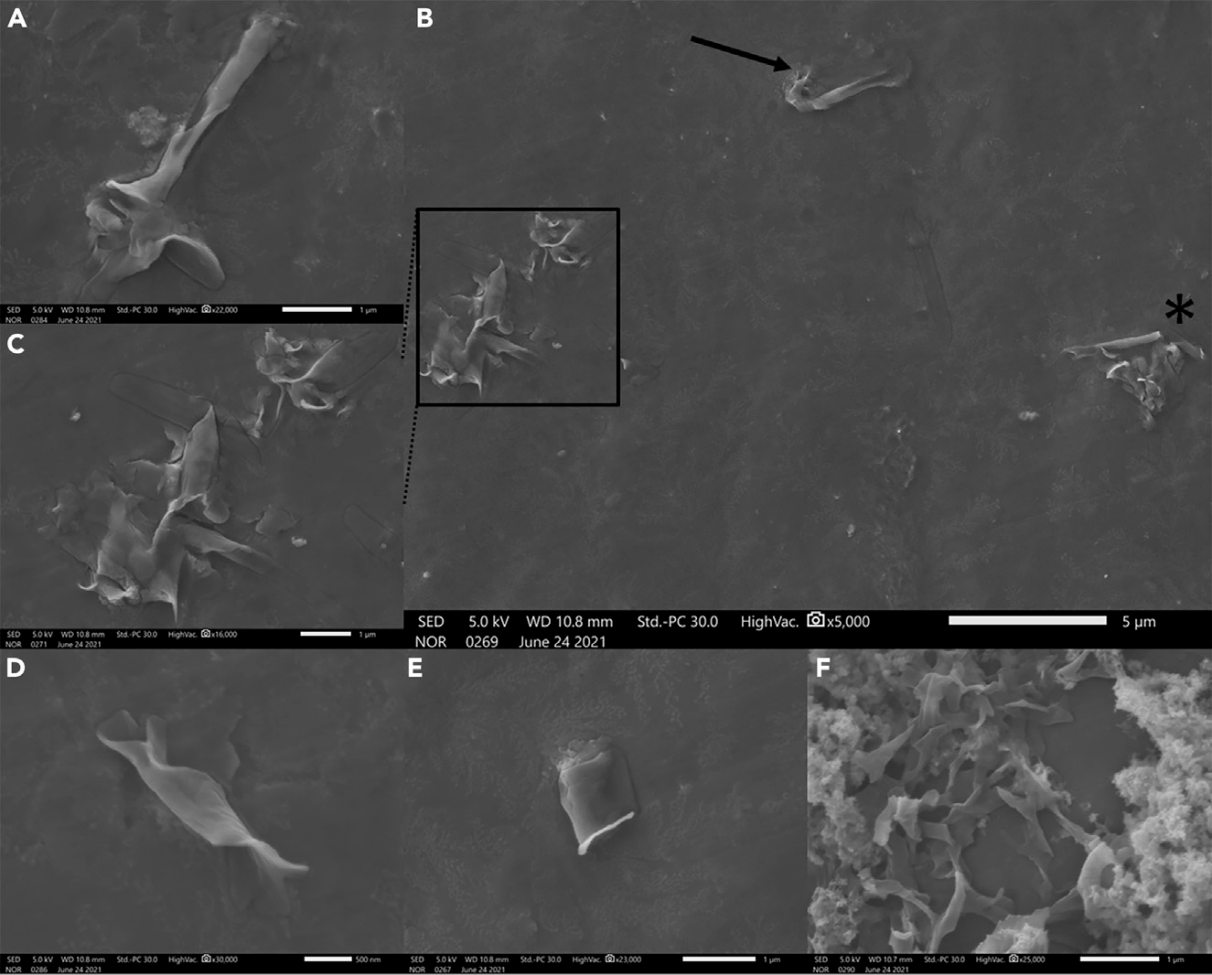
SEM characterization of aged nanopalm suspension for 120 days (A–F) (A) an intact nanopalm, (B) a completely deformed (squared area), partially deformed (∗), and an intact nanopalm (↘), (C) a zoomed-in view of the fully deformed nanopalm showing stable sheet and ribbons, (D) a folded sheet, (E) an unfolded sheet, and (F) stacked ribbons.

The aged suspension was further examined after 120 days to determine the stability of the various lipid structures. Stable quaternary structures of nanopalms were observed (Figure 6A) with their constituents; sheets and ribbons (Figure 6B and 6C). Folded (Figure 6D) or unfolded sheets (Figure 6E) and stacks of ribbons (Figure 6F) could also be detected. Nanoconfinement apparently stabilizes dynamic assemblies via strengthening the intramolecular bonding in a humid environment. Indeed, the high stability of formed lipid structures evokes exquisite control over the engineering rules adapted for the supramolecular assembly of highly stable nanomaterials.

## Discussion

Despite the extensive investigation of supramolecular self-assembly, understanding the rules governing this phenomenon remains challenging. Dynamic transition of one self-assembled structure to another occurs under certain conditions until reaching a hierarchical supramolecular structure of enhanced stability. The developed PPL approach permitted the assembly of lipids under nanoconfinement, which is, in our own opinion, a frontier to be routed. Upon adhesion of printed lipid micelles to PTT-primed substrate and following their rupture owing to hydrophobic interactions, the nanoconfinement effect facilitated the self-assembly of lipid molecules into discs when they are forced into close proximity (Figure 2A). These patterned discs (Figure 2A), transformed into various secondary structures such as sheets (Figure 2B), sheet& discs (Figure 2C), or ribbons (Figure S2) via intramolecular self-assembly mediated primarily by force and humidity in confined spaces. After their transfer in the solvent mixture (CHCl_3_/EtOH/DDW) via sonication and mechanical agitation (shaking), we speculate shredding of the generated lipid structures into smaller sheets and ribbons owing to the sonochemical effect while preserving their original shapes. EtOH could readily penetrate into the lipid bilayers and H-bond with the ester oxygens decreasing the interfacial tension of the lipid bilayers (Ly et al., 2002). In a similar manner, CHCl_3_ could partition into different sections of the lipid bilayers altering their inter-leaflet interactions (Reigada and Sagués, 2015). Such combined effect of the solvent mixture might cause structural curvature and downsizing. Comparably, ultrasonic treatment could induce local stresses via stable cavitation producing shear fields and thereby changing the bilayer curvature with breaking down the large lipid structures (Richardson et al., 2007). This mechanism is consistent with the effect of shear fields on producing smaller liposomes (Barba et al., 2014; Prieto et al., 2013; Woodbury et al., 2006). Complete evaporation of CHCl_3_& EtOH with continuous stirring triggered the formation of different supramolecular structures including cochleates (Figure 3A), discotic liquid crystals (Figure 3B), and nanopalms (Figure 3C). The hierarchical growth on the controlled self-assembly resulted in a highly ordered novel supramolecular structure through explicit manipulation of the printed lipid structures via PPL. Indeed, supramolecular chemistry could open new perspectives in materials science toward an area of supramolecular materials.

Analysis of aged suspension of nanopalms revealed the high stability of the resulting assemblies (sheets& ribbons) even after 120 days (Figure 6) in relation to the same assemblies formed in solution because of cationic bridging. The favorable intramolecular interactions within the confined nanoreactors could stabilize the resulting self-assembled structures. This gives rise to the intuitive intermolecular self-assembly on the mechanochemical treatment producing highly stable quaternary structures; the nanopalms. However, in the absence of nanoconfinement, structural disassembly might occur owing to the metastability of formed lipid structures (Nagarsekar et al., 2016; Richardson et al., 2007; Bozó et al., 2017). Reported supramolecular lipid structures such as cochleates and tubules originating from the unrestrained self-assembly in solution suffer from heterogeneity or high degrees of polydispersity (Nagarsekar et al., 2014, 2016; Fang, 2007). This lack of homogeneity could be overcome by PPL that could facilitate the patterning-mediated self-assembly via producing secondary structures of high uniformity. Indeed, engineering rules/design principles for generating homogenous supramolecular lipid structures can be proposed herein. It has been known that molecules in confined spaces behave distinctly from that in the bulk (Yang et al., 2008; Lopez-Fontal et al., 2018). Thereby, nanoconfinement of lipid molecules is the stellar engineering rule affecting their self-assembly spatially and temporally and thereby determining the structural shape, size, and stability. Other rules include controlled environmental parameters such as temperature and humidity as well as instrumental parameters including force and printing time that is mainly affected by the dwell time, pattern size, and z-piezo touch position. Such parameters govern the volume of delivered ink and its diffusivity affecting the shape, area, and uniformity of the resulting printed structures. Post patterning treatment is another rule of tremendous impact on the finally produced supramolecular nanostructures. Solvents, heat, and stirring all contributed to the folding, scrolling, and formation of highly uniform nanopalms. These cooperative and synergetic rules gave rise to the patterning-mediated self-assembly of the well-defined nanopalms. Indeed, the PPL-controlled generation of nanopalms through self-assembly offers a very powerful alternative to nanofabrication and nanomanipulation, bypassing the implementation of conventional procedures and providing a novel route to nanoscience and technology.

In conclusion, patterning of lipids via PPL offers a new route to study the generation of novel supramolecular quaternary lipid structures, the stability of which is determined via controlling the intramolecular self-assembly of lipid amphiphiles to form various secondary structures, including discs, sheets, and sheet & discs. Nanoconfinement promoted by PPL enables exquisite control over the dynamic assemblies of lipids by strengthening their intramolecular bonding in a humid environment. The fundamental knowledge gained from this work could enable the progressive build-up of more complex structures through hierarchical self-organization and in the process, allow achieving the control over the output supramolecular entity.

### Limitations of the study

Heterogeneity of the ink solution containing both micelles and vesicles can be further investigated to make the ink more homogenous consisting of only micelles. This would help improve the quantity and/or quality of generated nanopalms. Also, the proposed mechanism of nanopalm formation can be elucidated in depth to distinguish its capabilities over other lipid structures and enable the progressive build-up of more complex structures through hierarchical self-organization to achieve full control over the output supramolecular entity. Furthermore, all experiments were carried out using lab-scale instruments, thus; scalability of nanopalms shall be investigated to pave the route for industrial production of nanopalms for various applications.

## STAR★Methods

### Key resources table

**Table:** 

REAGENT or RESOURCE	SOURCE	IDENTIFIER
**Chemicals, peptides, and recombinant proteins**		

1,2-dioleoyl-sn-glycero-3-phosphocholine (DOPC)	Avanti® Polar Lipids	Cat# 850375P-25mg
1,2-dioleoyl-sn-glycero-3-phosphoethanolamine (DOPE)	Avanti® Polar Lipids	Cat# 850725P-25mg
Cholesterol (Chol)	Sigma Aldrich	Cat# C8667-100G
1-pentanethiol (PTT)	Sigma Aldrich	Cat# 8417490050
Pluronic F68	Specialty Products	Cat# 549920
Ethanol (EtOH)	Fisher Scientific, Ltd.	Cat# AC615110040
Chloroform (CHCl_3_)	LOBAChemie, Pvt. Ltd.	Cat# 00076

**Software and algorithms**		

SEM Operation	JEOL SEM (JSM-IT500HR), ASIA PTE. Ltd.	

**Other**		

Au substrates (2 × 2 cm^2^)	TERA-Print, LLC.	
PDMS pen arrays with 100 μm tip-spacing	TERA-Print, LLC.	

### Resource availability

#### Lead contact

Further information and requests for resources and reagents should be directed to and will be fulfilled by the lead contact, Ali H. Alhasan (aalhasan@kacst.edu.sa).

#### Materials availability

This study did not generate new unique reagents.

### Method details

#### Printing process

Au substrates (2 × 2 cm^2^) and PDMS pen arrays with 100 μm tip-spacing were purchased from TERA-Print, LLC.,USA. In a typical experiment, an Au substrate was primed with PTT-ethanolic solution (25 mM) to render its surface hydrophobic. The ink was prepared via gradual dissolution of DOPC (9.35 mg/mL), DOPE (8.85 mg/mL), and Chol (9.20 mg/mL) in a 50°C-preheated EtOH (0.33 mL) under continuous stirring till a transparent mixture of lipids was obtained. Subsequently, 50°C-preheated DDW (0.33 mL) was added drop-wise to the lipid mixture under stirring resulting in the formation of a micellar ink of lipids. Pluronic F68 (0.03 mM, 0.33 mL) was next added to the micellar ink in a drop-wise manner and the ink was subsequently kept under stirring at 50°C for 45 min. Based on this method of preparation, the ink is a heterogeneous mixture of vesicles and micelles. Mixed lipidic micelles (of either DOPE or DOPC) or polymeric micelles (of Pluronic F68) could form, whichfurther increases the heterogeneity of the ink. A PDMS array was plasma cleaned using Zepto plasma cleaner (Diener electronic-Germany, O_2_ plasma, 18 psi, 120 W, 220 s) directly before ink deposition via electrospraying using Spraybase® instrument (Spraybase®-Ireland, emitter inner diameter: 0.35 mm, flow rate: 1 mL/hr, distance from injector to collector: 10 cm, voltage: 8.4 kV, duration of electrospraying: 10 min). PPL experiments were carried out on the TERA-Fab M series, TERA-Print, LLC.,USA, operating at a temperature of 32 ± 2°C and a relative humidity (RH) of 92 ± 2%. Patterns of either 30x30 or 40x40 dots were created with a step size of 0.7 μm and a dwell time of 3 s. Printing force was varied utilizing 150, 230, 420, and 450 mN while keeping all other instrumental and experimental parameters constant.

#### Chemical treatment of printed substrates

Each printed substrate was dipped in a solvent mixture of CHCl_3_/EtOH/DDW (3:1:1) v/v% and sonicated for 10 min then mechanically agitated for 20 min, after which the substrate was removed and the solvent mixture containing the printed lipids was allowed to slowly evaporate at 35 ± 5°C under continuous stirring at 900 rpm. The obtained aqueous suspension was stirred for 48 hrs then filtered with 0.2 μm filter and the filtrate was finally stored in a 4°C fridge till use.

#### Scanning electron microscopy (SEM)

Micrographs were taken using JEOL SEM (JSM-IT500HR), ASIA PTE. Ltd., Singapore. In preparation for imaging, drops of samples were placed on SEM stubs, air dried, and coated with platinum (4 nm thickness) using the Auto Fine Coater (**JEC-3000FC),** JEOL, ASIA PTE. Ltd., Singapore.

#### Image processing

The obtained SEM images were processed using the SEM software (SEM Operation), wherein the ruler tool was utilized to quantitatively measure the outer diameters and end-to-end lengths of nanopalms. A line was drawn by tapping the start point of the target and dragging the line to the endpoint (Figure S6A). Once completely drawn, the measurement is displayed. This process was repeated for 123 nanopalms to calculate the average diameter/length and standard deviation (Figure S6B).

#### Transmission electron microscopy (TEM)

Micrographs were taken using JEOL TEM (JEM-1400Flash), ASIA PTE. Ltd., Singapore. In preparation for imaging, drops of samples were placed on TEM grids, stained with uranyl acetate and lead citrate, rinsed with DDW, and finally air-dried.

## Data Availability

This study did not generate new data and code.
