# Supplementary material for: Patterning-mediated supramolecular assembly of lipids into nanopalms
Source: iScience. 2022 Oct 13;25(11):105344. doi: 10.1016/j.isci.2022.105344 (PMC9619304; doi:10.1016/j.isci.2022.105344)
Supplement: Document S1. Figures S1–S6 [file mmc1.pdf]

iScience, Volume *25*

Supplemental information

Patterning-mediated supramolecular assembly of lipids into nanopalms

Samar A. Alsudir, Alhanouf Alharbi, Abdulaziz M. Almalik, and Ali H. Alhasan

Supplementary Information

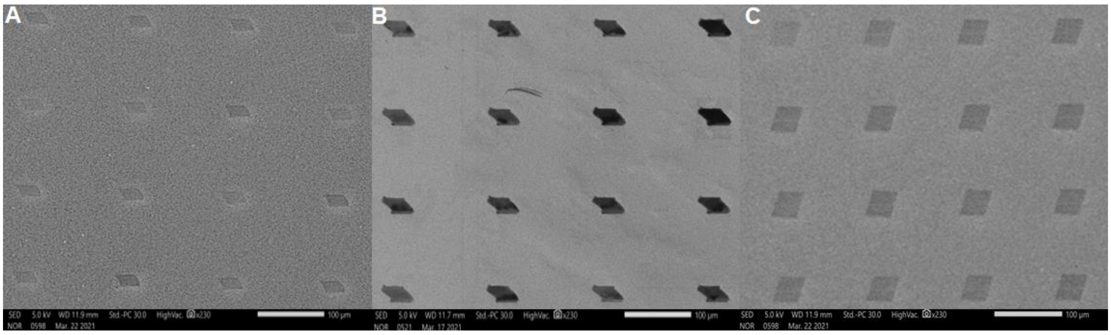

Figure S1: **SEM Characterization of the patterned lipid structures**. A) discs, B) sheets and C) sheet& discs, Related to Figure 2.

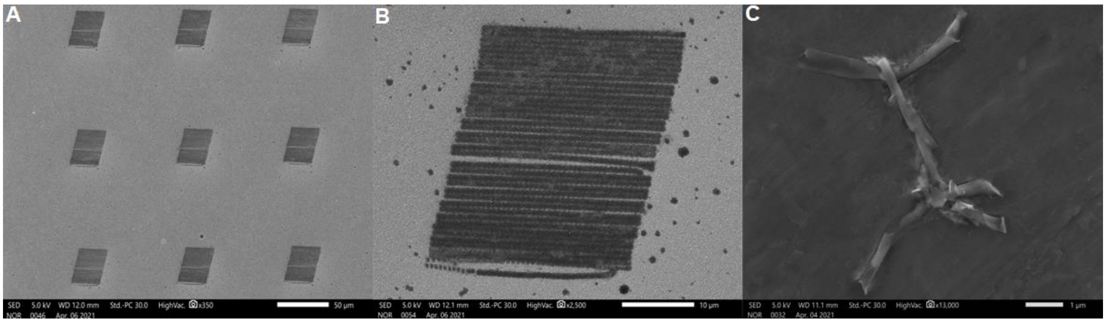

Figure S2: **SEM characterization of lipid ribbons.** Patterned lipid ribbons A,B) on PTT-primed Au substrate and C) in solution after emulsification, Related to Figures 2&3.

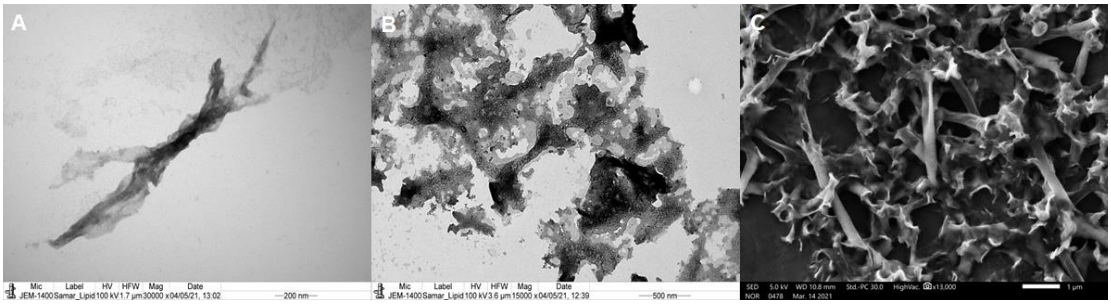

Figure S3: Characterization of lipid nanopalms. Micrographs of (A,B) TEM and (C) SEM, Related to Figure 3C.

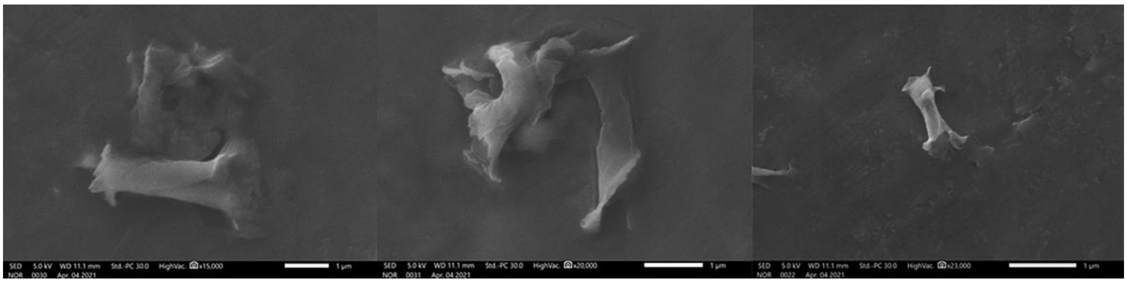

Figure S4: SEM characterization of sheet cochleates (SCs). Related to Figure 5.

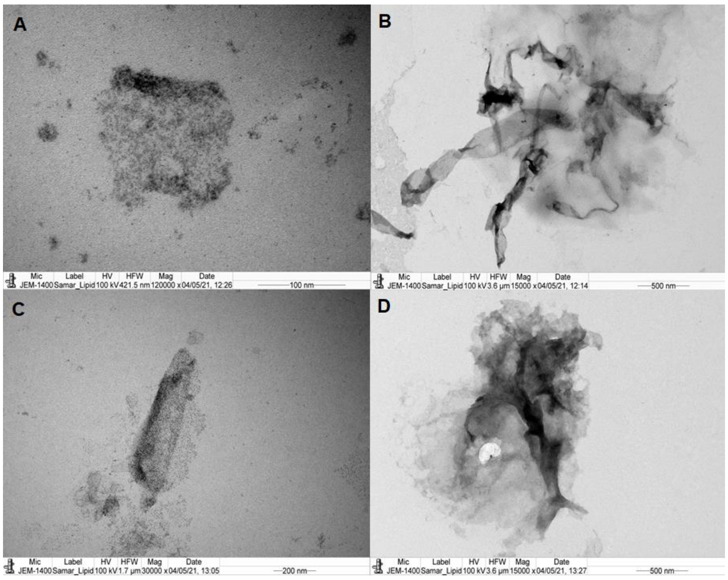

Figure S5: TEM characterization of nanopalm. The nanopalm constitutes of A) a sheet, B) ribbons, C) a sheet cochleate (SC), and D) ribbons wrapping a SC to form a nanopalm, Related to Figure 5.

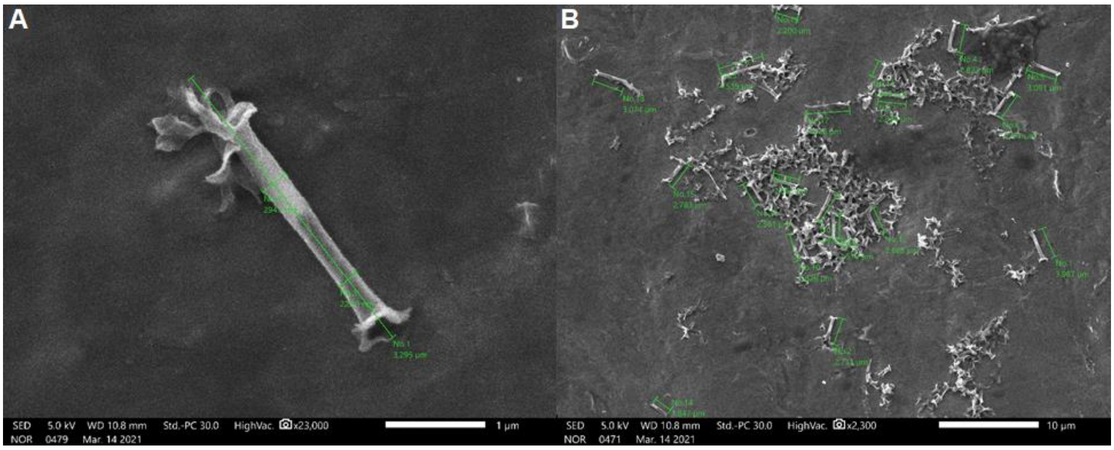

Figure S6: **Image processing of nanopalms.** SEM micrographs were analyzed to calculate the average diameter/ length; A) shows how the measurement of each nanopalm looks like, B) shows the quantifications of many nanopalms, Related to STAR Methods Image Processing.
